# Polynomial, Neural Network, and Spline Wavelet Models for Continuous Wavelet Transform of Signals

**DOI:** 10.3390/s21196416

**Published:** 2021-09-26

**Authors:** Andrey Stepanov

**Affiliations:** Institute of Magistracy, The Bonch-Bruevich Saint-Petersburg State University of Telecommunications, St. Petersburg 193232, Russia; sabarticle@yandex.ru

**Keywords:** wavelet, electroencephalogram, continuous wavelet transform, synthesis, mathematical model, polynomial, artificial neural network, spline

## Abstract

In this paper a modified wavelet synthesis algorithm for continuous wavelet transform is proposed, allowing one to obtain a guaranteed approximation of the maternal wavelet to the sample of the analyzed signal (overlap match) and, at the same time, a formalized representation of the wavelet. What distinguishes this method from similar ones? During the procedure of wavelets’ synthesis for continuous wavelet transform it is proposed to use splines and artificial neural networks. The paper also suggests a comparative analysis of polynomial, neural network, and wavelet spline models. It also deals with feasibility of using these models in the synthesis of wavelets during such studies like fine structure of signals, as well as in analysis of large parts of signals whose shape is variable. A number of studies have shown that during the wavelets’ synthesis, the use of artificial neural networks (based on radial basis functions) and cubic splines enables the possibility of obtaining guaranteed accuracy in approaching the maternal wavelet to the signal’s sample (with no approximation error). It also allows for its formalized representation, which is especially important during software implementation of the algorithm for calculating the continuous conversions at digital signal processors and microcontrollers. This paper demonstrates the possibility of using synthesized wavelet, obtained based on polynomial, neural network, and spline models, during the performance of an inverse continuous wavelet transform.

## 1. Introduction

Modern signal processing systems face more and more sophisticated requirements, particularly dealing with accuracy of information signs (features) in the signal. To meet this challenge, algorithms based on continuous wavelet transform (CWT) have become rather popular [1,2].

Wavelets are functions with a null integral value, localized along the time axis, capable of shifting along it and scaling [3].

The continuous wavelet transform [4] of the function *f*(*t*) is:

(1) $$W\left(a,b\right)=\frac{1}{\sqrt{\left|a\right|}}\int_{-\infty }^{+\infty }f\left(t\right)\bar{\psi \left(\frac{t-b}{a}\right)}dt,\;\;\;a,\;b\in R,\;a\neq 0,$$

where ψ(*t*)—wavelet, *a*—parameter that determines the size of the wavelet, *b*—sets shift along the time axis, *t*—time, *f*(*t*)—function, and the horizontal line indicates the complex fillet.

The result of a continuous wavelet transform is a matrix of wavelet coefficients calculated for different scale values *a* and shift of *b*:

(2) $$W\left(a,b\right)=\left[\begin{array}{cccc} W\left(a_{1},b_{1}\right) & W\left(a_{1},b_{2}\right) & \ldots & W\left(a_{1},b_{j}\right)\\ W\left(a_{2},b_{1}\right) & W\left(a_{2},b_{2}\right) & \ldots & W\left(a_{2},b_{j}\right)\\ & \;\;\;\;\;\;\;\;\;\ldots & & \\ W\left(a_{i},b_{1}\right) & W\left(a_{i},b_{2}\right) & \ldots & W\left(a_{i},b_{j}\right) \end{array}\right]$$

where *i*—the number of rows of the matrix (for the value *a*) and *j*—the number of columns of the matrix (for the value *b*).

Wavelet coefficients are commonly used to construct a wavelet-spectrogram, which can be used as a basis for carrying out time-frequency analysis of a signal.

Analyzing the formula (1), it can be noted that a significant influence on the results of continuous wavelet transform and the possibility of localization of graphoelements characteristic of the desired feature in the signal is the choice of maternal wavelet. The detection of a feature in the signal can be provided with a complete or significant coincidence of the fragment of the signal and the wavelet at a given shift *b* and scale *a*. At the same time, selection of scale *a* is related to main frequency of signal part:

(3) a=β/ω

where the constant β depends on the used wavelet, ω—main frequency of signal part.

For localization on the wavelet-spectrogram, the signal’s sought feature, it is possible to advise the selection of the closest to this feature’s form wavelet.

An existing set of wavelets does not provide the required variety of functions to define all types of features [5,6]. It can be supplemented with wavelets synthesized specifically for analysis of a definite signal’s type.

Synthesis will mean the procedure of obtaining maternal wavelet (briefly, the wavelet) ψ(*t*), satisfying the demands for continuous wavelet transform, and making a beginning for the family of functions ψ_*a*,*b*_(*t*) like:

(4) $$\psi_{a,b}\left(t\right)=\frac{1}{\sqrt{\left|a\right|}}\psi \left(\frac{t-b}{a}\right),\;a,b\in R,\;a\neq 0.$$

Synthesized wavelets, like all wavelets used in CWT have to meet the following requirements:

1. Meeting the validity condition [1,2]:

(5) $$C_{\psi }=\int_{-\infty }^{+\infty }\frac{{\left|\hat{\psi }\left(\omega \right)\right|}^{2}}{\left|\omega \right|}d\omega <+\infty$$

It is necessary for reversibility of continuous wavelet transform [4,7].

2. For practical-used wavelets, the tolerance condition (5) is equivalent [2]:

(6) $$\int_{-\infty }^{+\infty }\psi \left(t\right)dt=0,\;\mathrm{when}\;\int_{-\infty }^{+\infty }\left|t\psi \left(t\right)\right|dt<+\infty .$$

3. The wavelet ψ(*t*) must have a single norm [7,8,9]:

(7) ψ(t)=1

The contribution of this article can be summarized as follows:

1. We have proposed a modified algorithm for the synthesis of wavelets for continuous wavelet transform, which differs from the known ones by the guaranteed accuracy of approximation of the parent wavelet to a given sample (overlap match), with the possibility of a formalized representation of the wavelet. This algorithm allows one to solve an important practical problem. It allows synthesizing wavelets, which can later be used to perform continuous wavelet transform on an element base, where it is important to have a formalized representation of the basic function (microcontrollers, digital signal processors, graphics processors, etc.). This allows for the device-based computation of wavelet values directly and does not require additional memory to store them.
2. Neural network [10,11] and spline [12] wavelet models proposed by the author of this article have been investigated for the wavelet synthesis procedure for continuous wavelet transform. The results of their application are compared with the polynomial models and traditional wavelet families. The research uses real signals (fragments of an electroencephalogram) and test signals (to study the fine structure of the signal).
3. Computation of the inverse continuous wavelet transform using synthesized wavelets has been performed.

The main parts of the article are structured as follows. Section 2 presents similar and previous works. Section 3 proposes a modified wavelet synthesis algorithm. Also, we have made a comparison of the neural network and spline wavelet models proposed by the author of this article with polynomial wavelet models. Section 4 simulates the wavelet synthesis procedure and compares the results obtained for different models. Section 5 summarizes the conclusions and indicates the further research direction.

## 2. Related Works

Wavelets are widely used in signal analysis [1,2,3,4]. A significant part of wavelet signal analysis algorithms is based on the use of continuous wavelet transform [1,2,5,6,9]. The analysis of literary sources showed that wavelets are used in the analysis of signals such as electrocardiogram [13,14,15,16], electroencephalogram [6,17,18], seismic signals, signals from various sensors [1], etc.

At the same time, in most of the studies, traditional wavelet families such as Daubechies, Morlet, Mexican hat, etc., are used [1,2,6].

For a specific type of signal, one or another wavelet can be used. The choice of a wavelet, as a rule, is related to the characteristics of the signal. For example, for the analysis of a smooth signal, in most cases, the use of the Haar wavelet, which has a characteristic stepped form, is beside the purpose.

The results of continuous wavelet transform largely depend on the wavelet used; therefore, to obtain a better localization of information features on the wavelet spectrogram, the synthesis of wavelets adapted to a specific type of signal can be referred to [5,10,11,12].

Wavelet synthesis methods for continuous wavelet transform can be divided into 4 groups:

1. Methods for obtaining a wavelet by transforming another wavelet function. The main disadvantage of this method is, as a rule, the inability to obtain a wavelet of a given shape [1].
2. Methods based on the use of mirroring a part of a function from one quarter of the plane to another. This provides a zero integral value. This approach does not allow obtaining a formalized representation of the wavelet; its form is limited to a bipolar function. The adaptation of the wavelet to the signal is difficult.
3. Methods based on the use of an algebraic polynomial. In the wavelet synthesis procedure, an approximation of the exemplary signal fragment is performed. The advantages of this method are its simplicity, the possibility of obtaining a wavelet adapted to the signal, as well as a formalized representation of the function. The disadvantages include the limitations associated with the problem of ill-conditioning, as a consequence of the small degree of the polynomial and a significant deviation of the wavelet from the sample [5].
4. The method based on orthogonal functions makes it possible to obtain an adapted wavelet with a high accuracy of approximation to the sample, but there is no formalized representation of the wavelet. This imposes restrictions on the use of wavelets in portable applications, where it is required to calculate the wavelet values directly on the device (microcontroller, digital signal processor, etc.) [5].

Therefore, among the approaches considered, the most interesting is the polynomial method. It can also be concluded that it is necessary to develop a method for the synthesis of wavelets, which makes it possible to obtain a formalized representation of the wavelet and its exact approximation to the sample.

## 3. Modified Wavelet Synthesis Algorithm and Wavelet Models

In this paper, a modified wavelet synthesis algorithm for continuous wavelet transform is proposed. The algorithm makes it possible to meet all the previously described requirements. The proposed method differs from the known ones by the guaranteed accuracy of parent wavelet approximation to the sample, with the possibility of a formalized representation of the wavelet. This task uses neural network or spline models of the wavelet proposed by the author of this work [10,11,12].

### 3.1. Modified Wavelet Synthesis Algorithm

**Figure 1:**
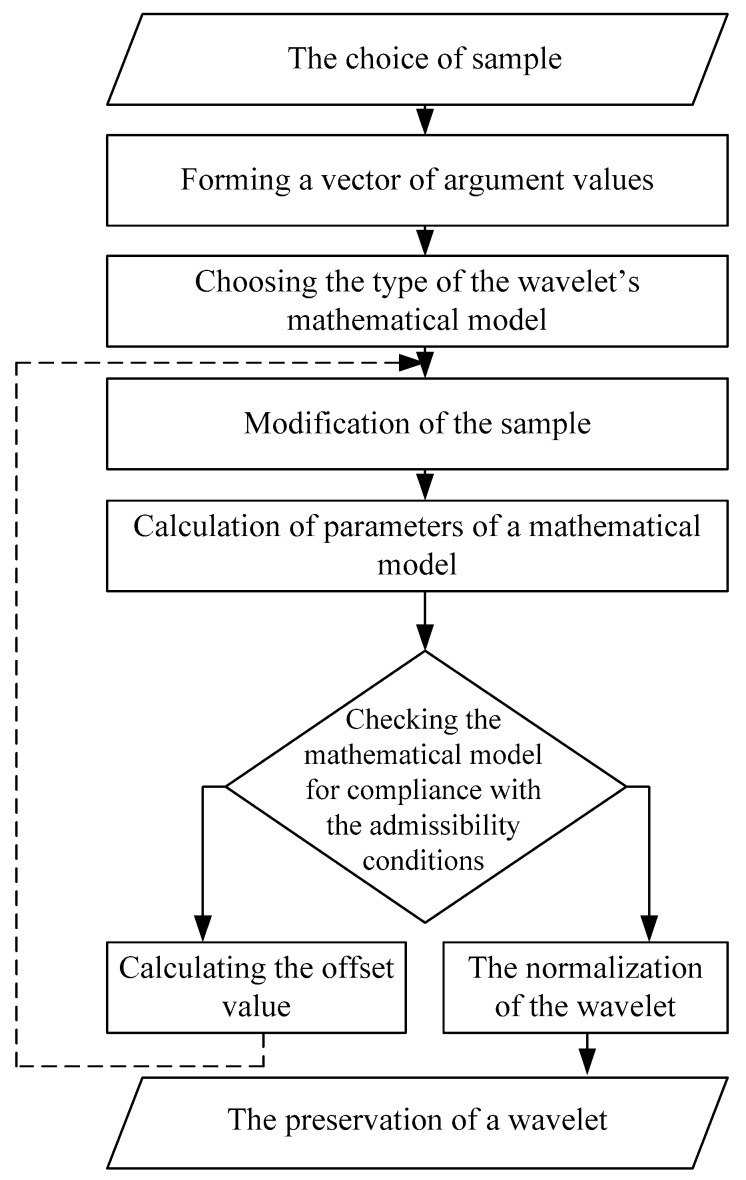
Modified wavelet synthesis algorithm for continuous wavelet transform of signals.

Let’s consider this algorithm (Figure 1):

(1) **The choice of sample.** At this stage, a fragment of the signal is selected. This sample will be used during the wavelet synthesis. It seems to be reasonable to use a signal’s fragment with a feature similar to the one needed to be determined in the signal’s analysis process. The fragment length is *N* samples.
(2) **Forming a vector of argument values.** The vector of values of an argument is formed in accordance with the length of the selected sample on the interval [0,1] with the number of elements *N* and in increments of 1/(*N* − 1).
(3) **Choosing the type of the wavelet’s mathematical model.** At this stage the mathematical apparatus that will be used to build a mathematical model of the wavelet was chosen.

The choice of the mathematical apparatus is made by the researcher on the basis of the requirements for the accuracy of approximation of the synthesized wavelet to the sample and the complexity of the mathematical model of the wavelet. The high accuracy of the wavelet approximation to the sample allows for high accuracy of the coincidence of the wavelet and the analyzed signal detail, which has a positive effect on the localizing ability of the wavelet spectrogram. The complexity of the wavelet mathematical model is limited by the computational capabilities of the element base, which is used to calculate the CWT. The studies carried out in this article allow us to give recommendations on the choice of a mathematical apparatus. They are summarized in the conclusion.

(4) **Modification of the sample.** As a rule, it is necessary to provide zero value of the wavelet at its extreme points, which may require the source fragment’s modification. In order to avoid sudden changes in the sample, it is proposed to multiply the selected fragment by a function of the form:

(8) $$w\left(n\right)=\{\begin{array}{c} f_{1}\left(n\right),\;n=\left[1,n_{1}\right];\;\;\;\;\;\\ 1,\;n=\left[n_{1}+1,\;n_{2}-1\right];\\ f_{2}\left(n\right),\;n=\left[n_{2},N\right];\;\; \end{array}$$

when *f*_1_(*n*) and *f*_2_(*n*) are increasing (from 0 to 1) and decreasing (from 1 to 0) functions; in the simplest case, they are linear functions.

(5) **Calculation of parameters of a mathematical model.** For a number of tasks, including the implementation of algorithms for calculating CWT on digital signal processors or microcontrollers [19,20], it is necessary to have a formalized representation of the wavelet’s mathematical model. It allows one to calculate the wavelet’s values at any time and for different scale. At this stage an approximation of a given type of mathematical model to the modified sample was made; the parameters are calculated, too.
(6) **Checking the mathematical model for compliance with the admissibility conditions.** In practice, the result was sufficient when the resulting function has a zero integral value can be considered. So, the following conditions are provided (6).
(7) **Calculating the offset value.** If the function does not satisfy the described condition, it is needed to make its modification. The way is to displace the sample along the axis statuses by increasing or decreasing each count by a constant. The initial value of this constant can be determined on the basis of the difference between the current integral value of the function and the desired one (zero). After that paragraphs 4–6 should be repeated. This procedure is iterative and for further automation it can be implemented as a program written, for example, in the MATLAB language.
(8) **The normalization of the wavelet.** Checking the wavelet for compliance condition looks like:

(9) $$\int_{-\infty }^{+\infty }{\left|\psi \left(t\right)\right|}^{2}dt=1$$

To obtain a parent wavelet ψ(t) satisfying the condition (9), it is necessary to multiply the function obtained in the previous steps $\psi_{f}\left(t\right)$ by the normalizing coefficient $M_{s}$:

(10) $$\psi \left(t\right)=M_{s}\psi_{f}\left(t\right)$$

when

(11) $$M_{s}=\frac{1}{\sqrt{\int_{-\infty }^{+\infty }\psi_{f}{}^{2}\left(t\right)dt}}$$

(9) **The preservation of a wavelet.** The result—wavelet’s mathematical model—can be saved on computer’s hard disk in the format, providing easy access when it is needed for use.

An important step in the wavelet synthesis procedure is the choice of the sample’s mathematical description method. It is necessary to ensure high accuracy of the obtained wavelet mathematical model’s approximation to an exemplary fragment. To estimate the accuracy of the approximation of the function, the minimum mean square deviation and the maximum deviation of the wavelet from the sample are used.

Let’s consider possible methods of obtaining such models.

### 3.2. Polynomial Wavelet Model

First of all, it should be noted that the term “polynomial wavelet model” is introduced by us in order to unify the terminology used in this work. In literature describing the procedure of obtaining a wavelet with application of algebraic polynomials [5] the term “adapted wavelet” is used. Despite the differences in terminology, the procedure itself is described very briefly and with an emphasis on modeling in MATLAB. Moreover, there are some restrictions on the choice of the maximum degree of the polynomial used, 6. They are installed in the math package itself, in the user’s graphical interface. It is also impossible to save the received wavelet in a formalized form. The new wavelet in MATLAB saves in two vectors (the values of the wavelet and the argument).

It is important to demonstrate a detailed description of the procedure for obtaining wavelets for continuous wavelet transform with the use of algebraic polynomial approximations. The information in the literature is not sufficient for the theoretical analysis of the results obtained wavelets; evaluation of possible restrictions and their application in practice without the use of particular mathematical software package is also difficult. The polynomial’s maximum degree was increased by avoiding the use of the proposed tools in the graphical user interface and writing our own script-file.

A polynomial wavelet model can be obtained by using the sample’s approximation with algebraic polynomial like *s*:

(12) $$F\left(x\right)=a_{0}+a_{1}x+a_{2}x^{2}+\ldots +a_{s}x^{s}$$

The main advantage of polynomial wavelet models is the relative ease of their obtaining. Moreover, the final result model usually has a small number of parameters—it simplifies further work with it.

However, the method of synthesis of wavelets for continuous wavelet transform based on the sample’s approximation by algebraic polynomials has a serious drawback. It can be noticed while trying to use a sample as the basis for a wavelet. The inability to get an exact approximation is meant. In order to increase the approximation’s accuracy it is necessary to increase the degree of the polynomial. As a result, some limitations caused by the problem of conditionality can be faced.

### 3.3. Wavelet Neural Network Model

Let’s introduce the concept of the neural network model of wavelets. The neural network model of wavelets will be understood as a mathematical expression describing the maternal wavelet ψ(*t*) on a given interval, which can be obtained during the modification of the sample and its mathematical representation with the help of artificial neural networks. Obviously, in this case, the number of parameters of the neural network determines the complexity of the model; at the same time, their values form and characteristics of the wavelet.

The author has proposed some neural network models of wavelets for continuous wavelet transform [10]. During the experiments [11], the possibility of applying the procedure for synthesis of wavelets for CWT multilayer perceptrons and neural networks based on radial basis functions (RBF-networks) was considered. This paper provides brief information about the results obtained, as well as some additions necessary for conducting further comparison.

As known [21], artificial neural networks can be considered universal approximators. It allows us to use them while different samples’ mathematical description in the synthesis of wavelets for continuous wavelet transform.

The simplest universal neural network approximators can be considered multilayer perceptrons and artificial neural networks based on radial basis functions. They can be used in the mathematical description of the sample in the wavelet synthesis procedure.

### 3.4. Spline Wavelet Model

Use of the interpolation sample by cubic splines at the stage of mathematical description is the basis for obtaining a spline model of the wavelet. During the process of interpolation, an interpolation spline was received. The procedure for obtaining such splines is known [22], therefore is not considered in details in this work. It should be noted that the values in the spline nodes must match the sample’s values.

In this case, the mathematical model of the wavelet for continuous wavelet transform looks like:

(13) $$\psi_{n}\left(t\right)=K_{l}\psi \left(t\right)\equiv K_{l}\psi_{i}\left(t\right)$$

where

(14) $$\psi_{i}\left(t\right)=a_{i,0}+a_{i,1}\left(t-t_{i}\right)+a_{i,2}{\left(t-t_{i}\right)}^{2}+a_{i,3}{\left(t-t_{i}\right)}^{3},\;\;t\in \left[t_{i},t_{i+1}\right],\;i=0,\ldots ,N-1,$$

where *N*—the sample’s length, *K_l_*—normalizing coefficient.

At the same time, it is important to ensure smooth docking of adjacent cubic polynomials in $\psi_{i}\left(t\right)$.

The values of the parameters $a_{i}$ are calculated for each range $\left[t_{i},t_{i+1}\right]$ while interpolating a sample. 

**Figure 2:**
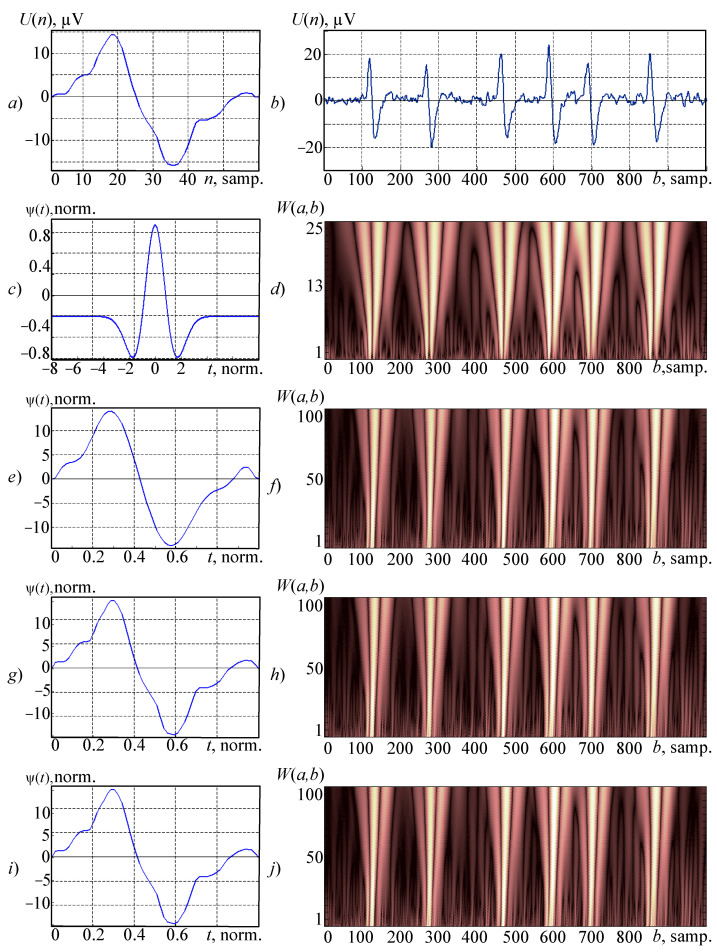
Results of research on synthesized wavelets: (**a**) the sample; (**b**) the analyzed signal; (**c**) the “Mexican hat” wavelet; the wavelet-spectrogram (**d**) that was obtained with its application; (**e**,**g**,**i**) the wavelets, obtained on the basis of polynomial, neural network, and spline models, respectively (before normalization); (**f**,**h**,**j**) wavelet-spectrograms obtained using synthesized wavelets (**e**,**g**,**i**), respectively.

The result (Figure 2i) completely coincides in overlaid with the sample (up to normalization). 

The advantage of the wavelet spline model is the guaranteed accuracy of the approximation of the obtained wavelet to the sample. The disadvantage is its high complexity. It is obvious that on each site $\left[t_{i},t_{i+1}\right]$ the wavelet will be set by 4 parameters, so the complexity of the model it is estimated as 4*N* + 1 parameter (taking into account the normalizing coefficient).

## 4. Results

Let us hold a set of experiments. A fragment of a real signal was selected (electroencephalograms) as the analyzed one and a test signal with a broken form.

An electroencephalogram (EEG) is a signal that can be registered from the surface of the human head and is the result of complex processes occurring in the brain [23,24,25]. Except the main EEG rhythms during the analysis, it is important to identify characteristic features of different pathological processes, as well as the artifacts (phenomena not related to brain activity). 

One of the most significant examples of this feature is eye artifact [23,24,25]. Its appearance is associated with eye movement during electroencephalographic research. As a rule, eye artifacts do not carry any useful information; they can create a hindrance during the right diagnosis and thus should be identified and excluded from the analysis.

In order to demonstrate the proposed neural network and wavelets’ spline models and a modified wavelet synthesis algorithm for continuous wavelet transform as a working sample, an EEG fragment with an eye artifact was used (Figure 2a). The selected fragment’s length is 0.24 s. (60 samples), and the sample’s signal frequency is 250 Hz. 

As the analyzed signal will select an EEG fragment of other record (Figure 2b), the length is 4 s. (1000 counts) with the same frequency as the sample’s. This fragment has 6 graph elements, usual for eye artifacts. During the analysis of such a signal on the wavelet spectrogram, 6 localized light sources areas should be allocated, corresponding to each individual graph element and value scale characteristic of the eye artifact’s main frequency (5 Hz). A chance to conduct a time-frequency analysis with high accuracy while features are localized in a signal on a wavelet spectrogram allows one to use synthesized wavelets during implementation of automatic analysis of electroencephalograms’ algorithms [10,11].

In order to evaluate the possibility of using traditional wavelet families while analyzing the EEG, get a wavelet spectrogram (Figure 2d) using the “Mexican hat” wavelet (Figure 2c). During the research of traditional wavelet families, it was found that this wavelet is the best one for EEG analysis. Even more—this wavelet is one of the few known wavelets having a formalized demonstration:

(15) $$\psi \left(t\right)=\frac{2}{\sqrt{3}}\pi^{\frac{-1}{4}}\left(1-t^{2}\right)e^{\frac{-t^{2}}{2}}.$$

The wavelet spectrogram shown in Figure 2d is necessary to perform a comparative analysis of traditional wavelet families with synthesized wavelets in the study of EEG. In this case, using exactly the wavelet “Mexican hat” (after formalized presentation) allows for more objective results. It should be noted that this wavelet-spectrogram was obtained in MATLAB. During its building, the scale values are arranged in ascending order from the bottom-up, which differs from the previously given wavelet matrix-coefficients (2). This order allows for responses to small details (high-frequency components) of the signal in the lower part of the wavelet- spectrograms and large details (low-frequency components) at the top parts of this graph. The maximum zoom value for this wavelet spectrogram was chosen to be *a* = 25. At level *a* = 12.5 (in the central part) the wavelet-spectrogram corresponds to the frequency of the part 5 Hz—the main frequency of the eye artifact. Analyzing this wavelet-spectrogram, it can be concluded that the eye artifacts on it are not localized well.

Let’s get wavelets based on the polynomial and—proposed in this paper—the neural network and spline models and demonstrate a continuous wavelet transform of the analyzed signal and compare the final wavelet-spectrograms.

### 4.1. Results of Polynomial Wavelet Models Application

The synthesis of a wavelet for the continuous wavelet transform based on the previously selected sample was made (Figure 2a). While approximating, an algebraic polynomial of the form was used (12). 

In practice were obtained the following results. Minimum value mean square deviations of synthesized wavelet (Figure 2e) from the sample (while being applied before normalization) are obtained using a polynomial of order *s* = 12 is 5.5 × 10^−4^ µV. Maximum deviation of the synthesized wavelet from sample size: 2.3434 µV (Table 1). Further increase in degree of the polynomial is limited by the problem of conditionality.

After normalization the synthesized wavelet can be represented:

(16) $$\psi \left(t\right)=K_{l}\left(a_{0}+a_{1}t+a_{2}t^{2}+\ldots +a_{12}t^{12}\right)$$

where *K_l_*—normalizing coefficient.

Due to the limited scope of this paper, the value of the normalizing the *K_l_* coefficient and as parameters are not given here.

This model is simple and has only 14 parameters (including normalizing coefficient).

During the construction of a wavelet spectrogram (Figure 2f), using the maximum scale value was selected for the synthesized wavelet *a* = 100. It makes it possible to get in its central part (*a* = 50) coefficients’ display corresponding to the main frequency of the eye artifact. The wavelet-spectrogram’s qualitative evaluation allows us to conclude that the localization of eye artifacts on it is better than in the case of application the “Mexican hat” wavelet. However, there are some obvious significant deviations of the wavelet obtained during synthesis from the sample. The necessity to apply other approaches to wavelets’ building models can be concluded; it will allow for a more accurate approximation of the wavelet to sample.

### 4.2. Results of Neural Network Wavelet Models Application

It is proposed to consider the possibility of using artificial neural networks when constructing wavelet models.

**Figure 3:**
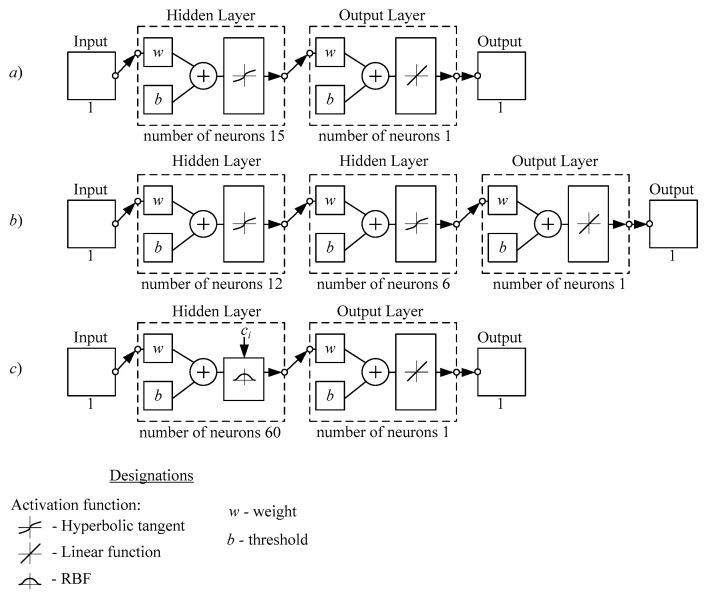
Artificial neural networks: multi-layer perceptron with one hidden layer (**a**), multi-layer perceptron with two hidden layers (**b**) RBF-network (**c**).

During the approximation of the previously selected sample (Figure 2a) the artificial neural networks shown in Figure 3 can be used. A training sample was formed to train neural networks. The simulation was performed in MATLAB.

Multi-layer perceptron with one hidden layer (Figure 3a) allows us to get a neural network wavelet model for CWT with a relatively small number of parameters (47, before the imposition of rationing). Just one hidden layer allows us to consider the model as simple, and it is a great advantage. The mathematical model of such a neural network is easier to implement in portable applications. The values of the wavelet should be calculated on the device itself (in order to save internal memory). As a disadvantage, it can be noted the significant deviation of the resulting wavelet from the sample (before the imposition of rationing). The minimum mean square deviation composed 0.0041 µV, the maximum deviation was 0.2663 µV (Table 1).

A multi-layer perceptron with two hidden layers (Figure 3b) allows us to get a neural network model describing the sample more accurately. As a drawback, it is necessary to note the increase of the model’s parameters (110, before the imposition of rationing) and the appearance of an additional layer. It is a significant complication. The minimum mean square deviation composed 0.0026 µV; the maximum deviation was 0.5356 µV (Table 1).

In both cases, the hyperbolic tangent function was studied for the neural network’s activation function (the logistics function allowed us to get close results). 

These neural networks were obtained in a practical way, and their parameters correspond to the minimum mean square deviation of the synthesized wavelet from the sample (before the imposition of rationing).

Figure 3c shows us an artificial neural network based on radial basis functions. This neural network allowed us to obtain minimum mean square deviation of the wavelet from the sample: 1.5762 × 10^−4^ µV and the maximum deviation of the wavelet from the sample: 0.6305 µV (Table 1). Thus, the RBF-network provides a higher accuracy of sample approximation compared to a multilayer perceptron. The accuracy of the approximation is the main advantage of this model; the key disadvantage is a significant number of its parameters (182). A large number of parameters leads to increased requirements for the computing base.

Figure 2g shows us a wavelet obtained from a neural network models using the given RBF-network (Figure 3c). After normalizing, a synthesized wavelet can be specified by an expression:

(17) $$\psi \left(t\right)=K_{l}\left(\overset{K}{\underset{i=1}{\sum }}w_{i}\varphi \left(t-c_{i}\right)\right),\;i=1,2,\ldots ,P,$$

when *P*—number of basis functions, *c_i_*—many centers, *K_l_*—normalizing coefficient.

However, this model has 182 parameters (taking into account the normalizing coefficient). It substantially exceeds the number of the polynomial model’s parameters and it is a significant disadvantage.

Figure 2h presents us a wavelet spectrogram obtained by using a wavelet based on this neural network model. Qualitative analysis of the wavelet-spectrogram allows us to note the accuracy localization of features on a wavelet-spectrogram. During the construction of this wavelet-spectrogram the maximum scale’s value is *a* = 100.

### 4.3. Results of the Wavelet Spline Model Application

When synthesizing a wavelet based on the selected sample, the resulting model will have 241 parameters (Table 1). Figure 2j shows us a wavelet-spectrogram obtained from using the synthesized wavelet and for the maximum value scale *a* = 100. It is necessary to note that the high accuracy of the display features on a wavelet-spectrogram look like localized light areas.

Results of the study of polynomial, neural network, and spline the models obtained in the analysis of EEG fragments are shown in Table 1.

As it can be concluded from the table, the most complicated is the spline model; however, it provides guaranteed accuracy of wavelet approximations to the sample.

**Figure 4:**
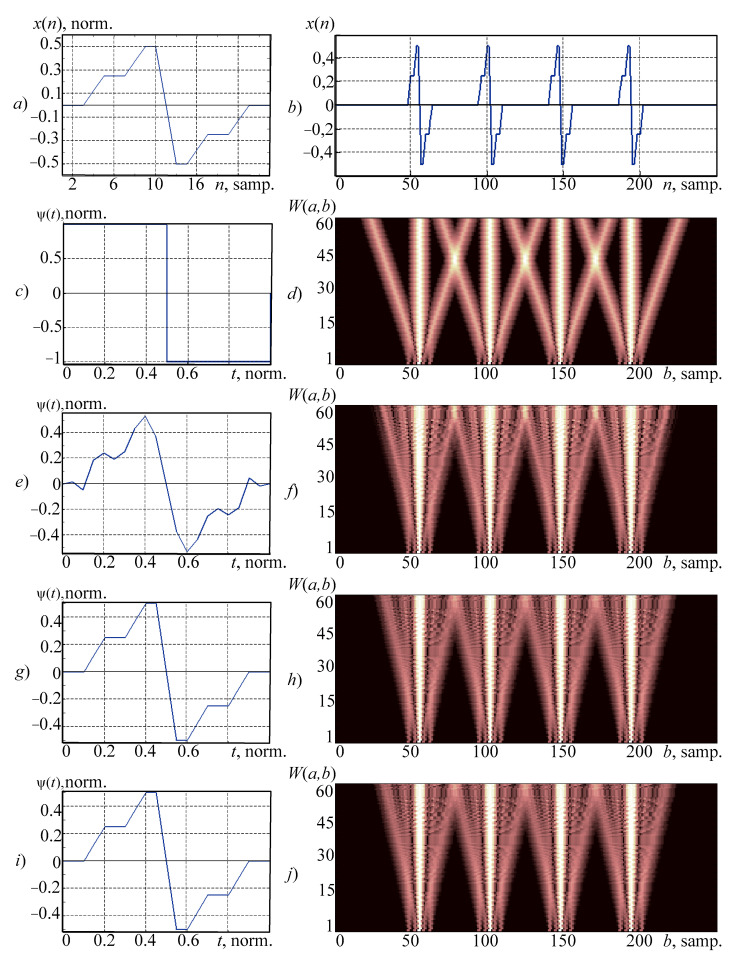
Results of research on synthesized wavelets: (**a**) the sample; (**b**) the analyzed signal; (**c**) the Haar wavelet; and the wavelet-spectrogram (**d**) that was obtained with its application; (**e**,**g**,**i**) the wavelets, obtained on the basis of polynomial, neural network, and spline models, respectively (before normalization); (**f**,**h**,**j**) wavelet-spectrograms obtained using synthesized wavelets (**e**,**g**,**i**), respectively.

In order to evaluate the possibility of using synthesized wavelets for analysis of the fine structure of the signal, it was proposed to consider the analyzed signal as a signal one (Figure 4b). This signal has a complex shape and obtained by serial connection of a broken signal’s fragments (Figure 4a) and zero segments.

Since the test signal has a broken type, in contrast to the previously considered smooth electroencephalographic signal, during the analysis by the traditional families of wavelets it can be advised to choose the Haar wavelet (Figure 4c). The result (Figure 4d) allows us to localize features, but in addition to the main one, the central cone also has external ones corresponding to the large value of coefficient. It can have a negative effect on the results of this type of signal’s automatic analysis.

Figure 4e demonstrates a wavelet derived from the polynomial models. In this case it was used an algebraic polynomial of the 12th order. According to the figure, its shape differs significantly from the sample. Figure 4f demonstrates a wavelet spectrogram obtained using this method. This wavelet-spectrogram is better for analysis such types of signals (than while using the Haar wavelet); however, in addition to the central cone it has two others with large coefficient values. Adjacent outer cones overlap each other. It can have negative effect on the results of this type of signal’s analysis.

Figure 4g,i presents the wavelets obtained while using a neural network (based on the RBF-network) and a spline wavelet’s model. Both wavelets match during the overlay with the sample (up to normalization). Ones obtained while using the wavelet-spectrogram (Figure 4h,j) have less pronounced external cones and they are more useful for automatic signal analysis than those ones discussed previously in this paper. Quantitative estimate model for this type of signal is given in Table 2.

As it can be concluded from the table, the most complicated is the spline model; however, it provides guaranteed accuracy of wavelet approximations to the sample. 

All synthesized wavelets can be used while performing a reverse continuous wavelet transform:

(18) $$f\left(t\right)=\frac{1}{C_{\psi }}\int_{-\infty }^{+\infty }\int_{-\infty }^{+\infty }\frac{1}{\sqrt{a}}W\left(a,b\right)\psi \left(\frac{t-b}{a}\right)\frac{dadb}{a^{2}}$$

**Figure 5:**
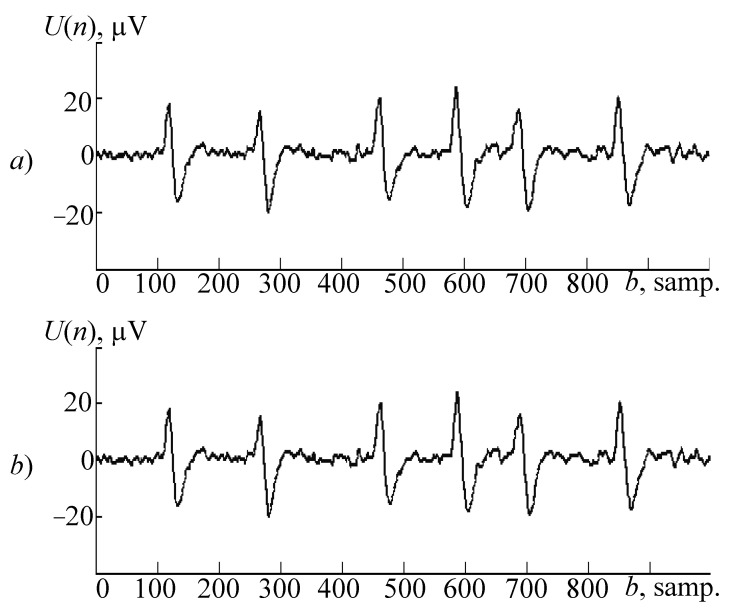
Results of the inverse continuous wavelet transform: (**a**) original and (**b**) restored signals.

Figure 5 shows the results of using wavelets obtained on based on spline models during inverse continuous wavelet transform. For this purpose the MATLAB program was used; the author modified m-file developed by H.-G. Stark [2]. During the restoration of a electroencephalography signal’s fragment scale’s coefficients, *a* = [1, 100] was used.

## 5. Conclusions

This paper’s key results:

An improved algorithm for the synthesis of wavelets for a continuous wavelet transform is proposed, which provides a formalized representation of the wavelet and a guaranteed accuracy of its approximation to the sample signal.

A neural network wavelet model for CWT allows us to get high accuracy of the wavelet-approximation to the sample’s signal. It provides a formalized representation of the wavelet.

A spline network wavelet model for CWT allows us to get high accuracy of the wavelet-approximation to the sample’s signal. It provides a formalized representation of the wavelet as well.

Theoretical studies of models are confirmed by the experiments’ results. An example fragment was a smooth electroencephalograms’ signal. During the analysis it was found that due to variability of graph elements corresponding to the same type (for example, eye artifact), the selection of complex mathematical models having a large number of parameters is not appropriate. This is why while defining large components in such signals it is possible to use polynomial wavelet models with fewer parameters than neural networks and splines. While analyzing signals in which the desired feature has a strictly fixed form it can be advised to use neural network and spline wavelet models, as they allow one to ensure high accuracy of such features’ localization. It was demonstrated in the analysis of signal’s fine structure; the example was a test signal of a broken type.

A study on the possibility of applying mathematical models and wavelets obtained from them in the reverse procedure continuous wavelet transform has been conducted. The final results confirm the reversibility of CWT while using synthesized wavelets.

Further research may include the following areas:

1. Application of the proposed mathematical models of wavelets and a modified wavelet synthesis algorithm in the development of an algorithm for automatic analysis of signals, for example, electroencephalograms. The author of this article has already achieved certain results in this direction.
2. Application of mathematical models of wavelets in time-frequency analysis of signals on the element base with low- and ultra-low power consumption. It should be noted, though, this will require optimization of algorithms for calculating the continuous wavelet transform. The author conducts research using MSP430 and STM32 microcontrollers.
3. Application of mathematical models of wavelets in the construction of activation functions of wavelet neural networks. The author of this article has already achieved certain results in this direction.

## Figures and Tables

**Table 1:** The results of mathematical wavelet models obtained for analysis of EEG fragments’ study.

Mathematical Model	Number of Models’ Parameters	Maximum Deviation, µV	Minimum Mean Square Deviation, µV
Polynomial	14	2.3434	5.5 × 10^−4^
Neuronet (perc. 1)	47	0.2663	0.0041
Neuronet (perc. 2)	110	0.5356	0.0026
Neuronet (RBF)	182	0.6305	1.5762 × 10^−4^
Spline	241	0	0

**Table 2:** The results of mathematical wavelet models obtained for analysis of broken signal’s study.

Mathematical Model	Number of Models’ Parameters	Maximum Deviation, Norm.	Minimum Mean Square Deviation, Norm.
Polynomial	14	0.1241	3.4467 × 10^−10^
Neuronet	65	1.5 × 10^−13^	4.2 × 10^−14^
Spline	84	0	0

## Data Availability

The data presented in this study are available on request from the corresponding author. The data are not publicly available due to the datasets involve unfinished research projects.
